# An open-label continuation trial of tocilizumab for familial Mediterranean fever with colchicine ineffective or intolerance: Study protocol for investigator-initiated, multicenter, open-label trial

**DOI:** 10.1097/MD.0000000000018328

**Published:** 2020-01-03

**Authors:** Tomohiro Koga, Naoko Hagimori, Shuntaro Sato, Shinpei Morimoto, Naoki Hosogaya, Chizu Fukushima, Hiroshi Yamamoto, Atsushi Kawakami

**Affiliations:** a Department of Immunology and Rheumatology, Division of Advanced Preventive Medical Sciences; b Center for Bioinformatics and Molecular Medicine, Nagasaki University Graduate School of Biomedical Sciences; c Nagasaki University Hospital, Clinical Research Center, Nagasaki, Japan.

**Keywords:** colchicine-resistant, FMF, IL-6, open-label, tocilizumab

## Abstract

**Background:**

Colchicine is the first-line treatment for familial Mediterranean fever (FMF), but secondary amyloidosis resulting from persistent inflammation is a concern in patients with colchicine-resistant or colchicine-intolerant FMF. Although tocilizumab (TCZ), which is a recombinant, humanized, anti-human interleukin 6 receptor monoclonal antibody, has been reported to prevent FMF attacks, the long-term safety and efficacy of TCZ on individuals with colchicine-resistant or colchicine-intolerant FMF have not been evaluated.

**Methods/design:**

In this investigator-initiated, multicenter, open-label trial, the long-term safety of TCZ will be evaluated in patients participating in a placebo-controlled, randomized, double-blind, parallel-group trial on colchicine-resistant or colchicine-intolerant FMF. The study will be conducted in 9 centers in Japan. After the evaluation and examination for 24 weeks in the preceding study, this trial will be started promptly. The trial will be completed by the time the drug is approved for FMF treatment in Japan. The primary endpoint is the incidence of adverse events, and the secondary endpoints include the number of FMF attacks, number of occurrences of accompanying symptoms during attacks, serum C-reactive protein and amyloid A levels, general evaluation by a physician (100 mm visual analog scale [VAS]), general evaluation by a patient (100 mm VAS), and body temperature.

**Discussion:**

The study is expected to obtain evidence regarding the long-term safety of TCZ as a potential new therapeutic agent for patients with colchicine-resistant or colchicine-intolerant FMF.

**Trial registration:**

This study was registered with the University Hospital Medical Information Network Clinical Trials Registry (https://upload.umin.ac.jp/cgi-open-bin/ctr_e/ctr_view.cgi?recptno=R000037116) as UMIN000032557 on May 30 2018.

## 1 Introduction

Familial Mediterranean fever (FMF) is the most common autoinflammatory disorder characterized by recurrent attacks of fever with arthritis, abdominal pain, skin rash, and/or serositis.^[1,2]^ In clinical practice, the therapy for FMF is introduced to prevent febrile episodes and to normalize levels of acute-phase reactants, such as C-reactive protein (CRP). The first choice of treatment is colchicine, which is effective in preventing FMF attacks and secondary amyloidosis development.^[3]^ However, 10% of FMF cases are refractory or resistant to colchicine.^[4,5]^ Canakinumab, an interleukin (IL)-1 beta-inhibitor, is considered for patients with colchicine-resistant or colchicine-intolerant FMF, but evidence of the efficacy or safety of this treatment in Japanese patients with FMF is limited.

We have previously reported that IL-6 is the most important cytokine to distinguish between attack and remission in patients with FMF in addition to those with FMF attacks and to healthy individuals.^[6]^ These findings suggest that IL-6 may be useful as a biomarker for FMF and that tocilizumab (TCZ), which specifically inhibits IL-6 signal, may be useful as a therapeutic agent. To confirm the long-term safety and efficacy of TCZ on individuals with colchicine-resistant or colchicine-intolerant FMF, we are currently recruiting patients with FMF who completed a phase III, investigator-initiated, multicenter, double-blind, randomized, parallel-group trial.^[7]^ Herein, we describe the final protocol (version 1.3; July 12, 2019) for this study. The results of this study are expected to provide evidence regarding the long-term safety of TCZ in the treatment of patients with colchicine-resistant or colchicine-intolerant FMF.

## 2 Methods/design

### 2.1 Study design

The present study design is in accordance with the Standard Protocol Items: Recommendations for Interventional Trials and Consolidated Standards of Reporting Trials 2010 guidelines.^[8,9]^ This is an open-label, investigator-initiated, multicenter study on the efficacy and safety of TCZ compared with placebo in patients with colchicine-resistant or colchicine-intolerant FMF.

The study will be conducted at 9 centers in Japan. The study is registered on the University Hospital Medical Information Network Clinical Trials Registry (https://upload.umin.ac.jp/cgi-open-bin/ctr_e/ctr_view.cgi?recptno=R000037116) as UMIN000032557. We will conduct the study in accordance with the principles of the Declaration of Helsinki^[10]^ and the Japan good clinical practice. The local ethics committee of each center will approve the study.

### 2.2 Participant recruitment

Participants will be recruited at the Nagasaki University Hospital, Kyushu University Hospital, Kyoto University Hospital, Yokohama City University Hospital, Chiba University Hospital, Kanazawa University Hospital, Shinshu University Hospital, Fukushima Medical University, and Hokkaido University Hospital. Participants will be provided with an explanation regarding the study by their treating pediatrician/rheumatologist and clinical research coordinator (CRC) and asked to voluntarily sign an informed consent form before their participation.

### 2.3 Inclusion criteria

The inclusion criteria include the following:

(1) completed the 24-week treatment with an investigational drug in the preceding trial (UMIN000028010) and
(2) obtained a thorough explanation of the contents of explanatory documents and other matters concerning clinical trials, understood the contents thereof, and provided written consent based on their free will to participate in this trial.

### 2.4 Exclusion criteria

The exclusion criteria are as follows: breastfeeding, pregnancy, or planning for pregnancy; obvious infection within 4 weeks before the study and considered inappropriate by an investigator or clinical trial physician; history of hypersensitivity to the components of TCZ; history of interstitial pneumonia and judged inappropriate by the investigator or clinical trial physician; routine use of corticosteroids (excluding topical therapy, such as external preparations) due to diseases other than FMF; and judged by the clinical investigator or clinical trial physician as inappropriate for any other reason.

### 2.5 Study protocol

**Figure 1:**
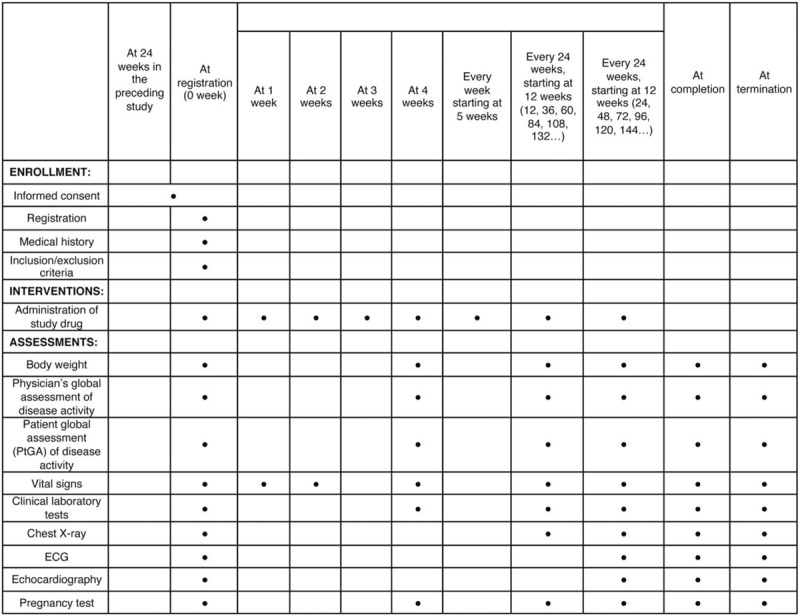
Treatment schedule and outcome measures. ECG = electrocardiogram.

A clinical trial physician will explain the study protocol to each patient with colchicine-resistant or colchicine-intolerant FMF who have completed 24 weeks of treatment in the preceding study. If the patient's consent is obtained, a clinical trial physician will perform the observation/examination at the time of registration based on the description in Figure 1. According to the inclusion and exclusion criteria, the CRC will fax a registration form to the registration center. A patient diary will be offered to each participant on the visit date at week 0 of the study. The participants will be instructed to complete the patient diary, including daily body temperature and accompanying symptoms, such as headache, arthritis, chest pain, back pain, and abdominal pain.

After the visit date (ie, the initial investigation day of the investigational drug), the investigators will continue to administer the investigational drug and conduct necessary examinations and surveys in accordance with the schedule presented in Figure 1. The observation/examination will continue until TCZ is approved for FMF treatment in Japan. If the decision is made to discontinue the development of the investigational drug for FMF treatment, the study will be immediately terminated.

### 2.6 Adverse events

All SAEs occurring between the signing of the informed consent and the end of the trial will be recorded. An SAE is defined as any untoward medical event that occurs at any dose, results in death, is life-threatening, requires inpatient hospitalization or prolongation of existing hospitalization, results in persistent or significant disability or incapacity, or causes a congenital anomaly or birth defect.

The study design includes an independent data monitoring committee that will review the ongoing safety data in an unblinded manner in accordance with the Standard Operating Procedures for Clinical Trials, Japan Medical Association (http://www.jmacct.med.or.jp/).

### 2.7 Outcome

The primary endpoint is the incidence of adverse events. The secondary endpoints are efficacy and safety categories.

### 2.8 Efficacy

We will evaluate the efficacy of the investigational drug based on the following parameters: number of fever attacks, number of occurrences of accompanying symptoms during attacks, serum CRP, and serum amyloid A (SAA) levels, general evaluation by a physician (100 mm visual analog scale [VAS]), general evaluation by a patient (100 mm VAS), and body temperature.

### 2.9 Safety

The safety evaluation indices of this clinical trial are as follows: adverse events (adverse event incidence rate, serious adverse event incidence rate, and side effect incidence rate), clinical examination (hematological examination, blood biochemical examination, and urinalysis), all medically important indicators (physical findings, vital signs, electrocardiogram results, echocardiographic findings, etc), and pharmacodynamic assessment, including the measurement of the serum TCZ and soluble IL-6 receptor levels.

### 2.10 Data collection and management

Appropriate and authorized persons (investigators, clinical trial physicians, and clinical trial collaborators) prepare a case report form (CRF). All data recorded in the CRF must be consistent with the original material unless the data recorded directly in the CRF are used as the source material. According to the schedule presented in Figure 1, the investigator will collect data at each visit during the study.

The investigators will be provided access to an online, web-based, electronic data-capture system. Only the investigator will be able to enter and modify data in the electronic CRF (e-CRF). All study findings and documents will be regarded as confidential. Patients will be identified on the e-CRF by their patient number and/or birth date, not by name. Confidentiality of the documents that identify the patient must be maintained by the investigator so that the anonymity of the participants is ensured.

During the study, a sponsor-investigator will perform regular site visits to review protocol compliance, conduct source data verification, assess drug accountability and management, assess laboratory procedures, and ensure that the study is being conducted according to pertinent regulatory and protocol requirements.

### 2.11 Statistical analysis

We define the population to be analyzed as all subjects who received at least 1 dose of TCZ throughout the preceding study. All TCZ-treated populations are summarized using summary statistics. The safety and tolerability analyses will be based on this analysis set. We will replace adverse events with the corresponding MedDRA code and tabulate the number of expression cases for each event defined by the system organ class and preferred term. Among these, in cases that cannot be denied causally, the number of patients and the number of events are separately counted as side effects. We will calculate the rate of adverse events (times/patient-year), rate of SAEs (times/patient-year), and rate of adverse reactions (times/patient-year) and their bilateral 95% confidence intervals. We will prepare charts showing changes in laboratory values, summary statistics, and tables. To determine the rate of continuation of this trial, we will calculate the proportion of subjects who are able to continue the trial at each time point. These analyses will be conducted separately from the preceding study and the present study as necessary.

For efficacy, summary statistics will be calculated at 0, 4, 12, and 12 weeks intervals, thereafter using the following endpoints: number of febrile episodes, occurrence of accompanying symptoms during attacks, serum CRP and SAA levels, results of a general evaluation by a physician (100 mm VAS), results of a general evaluation by a patient (100 mm VAS), and change in body temperature.

Importation of data related to this clinical trial is performed using the SAS software version 9.2 or higher. The dataset creation for the statistical analyses will be performed using the SAS software. Statistical tests will be 2-sided, and *P*-values < .05 will be considered as significant for the primary endpoint.

## 3 Discussion

The goal of FMF treatment is to prevent not only acute attacks but also the development and progression of amyloidosis by minimizing subclinical inflammation between attacks. Thus, the use of intermittent colchicine only for the treatment of acute episodes of FMF is not recommended as it does not prevent the development of amyloidosis due to mild inflammation that can occur during asymptomatic periods.^[11]^ TCZ inhibits IL-6, which is important in the pathogenesis of FMF, but does not eradicate FMF. A long-term use of TCZ is desirable to prevent the progression of amyloidosis, and verifying the long-term safety of TCZ in patients with FMF is imperative.

TCZ has already been approved for rheumatoid arthritis (RA) treatment in Japan in 2008, and the safety profile of intravenous TCZ has been previously shown. This study reveals that mortality rates and incidence rates of serious infections, malignancies, gastrointestinal perforations, and severe cardiac dysfunction were consistent for 3 years.^[12]^ The TCZ used in this study is a subcutaneous injection. A previous study showed that the long-term efficacy and safety of subcutaneous TCZ was maintained and comparable to that of intravenous TCZ.^[13,14]^ These data indicate the long-term safety of TCZ use in patients with RA in actual clinical settings. Compared with patients with FMF, patients with RA are more likely to be administered immunosuppressants, such as methotrexate and glucocorticoids. Accordingly, it could be safer to use TCZ in patients with FMF than those with RA.

The primary endpoint of this study is the definition of the safety profile, but long-term efficacy will also be assessed as a secondary endpoint. Although there are some studies that show the efficacy of TCZ in patients with FMF,^[15,16]^ the factors for the rate of continuation of TCZ in patients with FMF are unclear, and it is important to identify the rates of adverse events and secondary failures for TCZ. Additionally, this study will include patients with FMF without amyloidosis, which may confirm the long-term protective effect of TCZ on the development and progression of amyloidosis.

Therefore, this trial will evaluate the long-term safety and efficacy of TCZ in patients with colchicine-resistant or colchicine-intolerant FMF. It will contribute to the identification of the effects of long-term TCZ use on the development of amyloidosis. These findings may support the evidence of IL-6 targeted therapy in patients with severe FMF.

## Acknowledgments

The authors would like to thank our colleagues and staff at the Rheumatology Department of Nagasaki University Hospital for their support.

## Author contributions

TK, SS, HY and AK are responsible for conceiving and designing the trial, planning data analysis, drafting the manuscript, making the final decision to terminate the trial, and approving the final manuscript. TK, NH, and CF will participate in data collection and are in charge of recruitment and treatment of patients. SS and SM are responsible for planning data analysis and analyzing the data resulting from the trial. All authors will have access to the interim results as well as the capacity to discuss, revise, and approve the final manuscript.
